# Subcutaneous adipose tissue sclerostin is reduced and Wnt signaling is enhanced following 4‐weeks of sprint interval training in young men with obesity

**DOI:** 10.14814/phy2.15232

**Published:** 2022-03-21

**Authors:** Nigel Kurgan, Hashim Islam, Jennifer B. L. Matusiak, Bradley J. Baranowski, Joshua Stoikos, Val A. Fajardo, Rebecca E. K. MacPherson, Brendon J. Gurd, Panagiota Klentrou

**Affiliations:** ^1^ Department of Kinesiology Brock University St. Catharines Ontario Canada; ^2^ Centre for Bone and Muscle Health Brock University St. Catharines Ontario Canada; ^3^ School of Health and Exercise Sciences University of British Columbia Okanagan Kelowna British Columbia Canada; ^4^ Department of Kinesiology Queens University Kingston Ontario Canada; ^5^ Department of Health Sciences Brock University St. Catharines Ontario Canada

**Keywords:** bone–adipose tissue crosstalk, sclerostin, sprint interval training, Wnt signaling

## Abstract

Sclerostin is a Wnt/β‐catenin antagonist, mainly secreted by osteocytes, and most known for its role in reducing bone formation. Studies in rodents suggest sclerostin can also regulate adipose tissue mass and metabolism, representing bone–adipose tissue crosstalk. Exercise training has been shown to reduce plasma sclerostin levels; but the effects of exercise on sclerostin and Wnt/β‐catenin signaling specifically within adipose tissue has yet to be examined. The purpose of this study was to examine subcutaneous WAT (scWAT) sclerostin content and Wnt signaling in response to exercise training in young men with obesity. To this end, 7 male participants (BMI = 35 ± 4; 25 ± 4 years) underwent 4 weeks of sprint interval training (SIT) involving 4 weekly sessions consisting of a 5‐min warmup, followed by 8 × 20 s intervals at 170% of work rate at VO_2peak_, separated by 10 s of rest. Serum and scWAT were sampled at rest both pre‐ and post‐SIT. Despite no changes in serum sclerostin levels, we found a significant decrease in adipose sclerostin content (−37%, *p* = 0.04), an increase in total β‐catenin (+52%, *p* = 0.03), and no changes in GSK3β serine 9 phosphorylation. There were also concomitant reductions in serum TNF‐α (−0.36 pg/ml, *p* = 0.03) and IL‐6 (−1.44 pg/ml, *p* = 0.05) as well as an increase in VO_2peak_ (+5%, *p* = 0.03) and scWAT COXIV protein content (+95%, *p* = 0.04). In conclusion, scWAT sclerostin content was reduced and β‐catenin content was increased following SIT in young men with excess adiposity, suggesting a role of sclerostin in regulating human adipose tissue in response to exercise training.

## Graphical abstract

In this study, we examined sclerostin content in serum and subcutaneous adipose tissue both pre‐ and post‐exercise training in humans with obesity. Our data support that sclerostin decreases while Wnt/β signaling, the pathway sclerostin antagonizes, increases in adipose tissue fol. These results provide observational evidence for an endocrine role of sclerostin in humans and suggest a role of sclerostin/Wnt signaling in exercise induced adipose tissue adaptations.

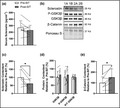

## 1 INTRODUCTION

Exercise training offers protection against several metabolic disorders, including obesity and insulin resistance/type II diabetes. Adipose tissue adaptations to exercise training appear to play a central role in mediating protection against metabolic dysfunction (Stanford & Goodyear, 2016; Stanford et al., 2015) and chronic disease. These adaptations, which have mainly been studied in murine models, include reduced adipogenesis (Gollisch et al., 2009; Sakurai et al., 2010), alterations in the composition of resident immune cells (Soltani et al., 2020) (e.g., adipose tissue macrophages (Macpherson et al., 2015)), changes in the expression and release of adipokines (e.g., decreased circulating pro‐inflammatory TNF‐α Khalafi & Symonds, 2020; Sardeli et al., 2018), mitochondrial biogenesis (McKie & Wright, 2020), and white adipose tissue browning (e.g., increased expression of uncoupling protein (UCP) 1) (Boström et al., 2012; Cao et al., 2011; Lehnig et al., 2019; Stanford et al., 2015; Sutherland et al., 2009; Trevellin et al., 2014). Studies examining the role of various muscle derived proteins (i.e., myokines) in regulating several adipose tissue adaptations to exercise have highlighted the importance of tissue crosstalk during exercise. In contrast, less is known regarding bone‐derived proteins (i.e., osteokines) in regulating adipose tissue at rest or in response to exercise training.

Studies in murine models have shown that ablation of osteocytes, bone's mechanosensory cells, leads to a loss of white adipose tissue (Sato et al., 2013; Sato & Katayama, 2015), suggesting a possible role of osteocyte‐derived osteokines in regulating whole body metabolism and fat mass. Osteocytes coordinates bone modeling and remodeling by secreting factors that regulate the cells that form (osteoblasts) and resorb (osteoclasts) bone (Bonewald, 2011). One factor critical to this regulation and secreted mainly by the osteocyte is the glycoprotein sclerostin, which inhibits the canonical Wnt/β‐catenin signaling pathway (Lin et al., 2009; Spatz et al., 2015). Within bone, the canonical Wnt/β‐catenin signaling cascade inhibits GSK3β by disassembling the destruction complex, preventing the proteolytic degradation of β‐catenin, which increases its accumulation, subsequently activating Wnt target genes and leading to an increase in bone formation. Thus, inhibiting sclerostin, with the use of sclerostin neutralizing antibodies, has been used as a therapeutic target for treating low bone mass (Langdahl et al., 2017). In mice, genetic deletion of *sclerostin* or inhibiting sclerostin via receptor antagonism or neutralizing antibody increases bone mass while also improving glucose and lipid homeostasis (e.g., increase in glucose and insulin tolerance and reduced serum fatty acids) (Kim et al., 2017b; Kim et al., 2019). Along with these changes, reductions in adipose tissue mass, and adipocyte cross‐sectional area can also be observed with sclerostin inhibition, ultimately conferring resistance to a high‐fat obesogenic diet. These changes are a consequence of augmented Wnt signaling, which has been shown to inhibit adipocyte maturation and differentiation (Kim et al., 2021; Li et al., 2008; Steinhart & Angers, 2018).

There is also clinical evidence supporting a role of sclerostin in regulating metabolism and fat mass in humans. Serum sclerostin has been shown to be elevated in individuals with prediabetes and correlates with circulating levels of low‐density lipoprotein cholesterol (Urano et al., 2012) and insulin resistance in skeletal muscle, liver, and adipose tissue (Daniele et al., 2015; Stanik et al., 2019; Wędrychowicz et al., 2019). Sclerostin also appears to be associated with age and fat mass (Amrein et al., 2012; Urano et al., 2012). Additionally, higher plasma sclerostin is associated with lower Wnt signaling gene expression in WAT of prediabetic males (Janssen et al., 2019), suggesting sclerostin is having an endocrine‐like effect on adipose tissue and increases in individuals with dysregulated metabolism. Interestingly, circulating sclerostin decreases in response to moderate intensity exercise training in older adults (Ardawi et al., 2012; Hinton et al., 2017; Janik et al., 2018) and Wnt target genes have been shown to increase in scWAT of mice following exercise training (Stanford et al., 2015). Taken together, these findings suggest a role of sclerostin and Wnt signaling in regulating adipose tissue adaptations in response to exercise training. However, no study has examined sclerostin protein content within human scWAT at rest or in response to exercise training, which is required to provide initial evidence for a role in regulating adipose tissue in response to exercise training prior to studies that examine the mechanistic significance.

The primary purpose of this pilot study was to measure circulating sclerostin and examine scWAT for changes in sclerostin content and Wnt signaling (β‐catenin accumulation) following 4 weeks of sprint interval training (SIT) in young men with obesity. By examining sclerostin content in human scWAT, we aim to provide initial observational evidence that sclerostin acts as a bone derived regulator of adipose tissue in response to exercise training. SIT was chosen based on previous studies finding similar fat oxidation over 24 h post‐exercise compared to endurance exercise (Hazell et al., 2012) and reductions in circulating sclerostin occur in similarly aged participants following low‐volume SIT (Sansoni et al., 2018). Also, given individuals with higher adiposity have been shown to have higher levels of circulating sclerostin (Amrein et al., 2012) and that diet‐induced obese murine models have higher bone sclerostin expression (Baek et al., 2014), we recruited young men with excess adiposity as this group was expected to have the largest response. We hypothesize that there will be a reduction in scWAT sclerostin and increased β‐catenin accumulation through upregulation of canonical Wnt signaling following SIT. Furthermore, a secondary objective of this study was to reproduce findings observed in murine studies, which include examining markers of mitochondrial biogenesis (PGC1α, cytochrome c, COXIV, and UCP1), which have been shown to increase in rodents scWAT, while findings in humans have been inconclusive (Stanford & Goodyear, 2016).

## 2 METHODS

### 2.1 Participants

Eight young men (25 ± 4 years old) were recruited to partake in this study. Participants were included if they were between the ages of 18–30, not involved in an exercise training program at the time of enrolment, performing no more than 2–3 weekly sessions (150 min) of self‐reported moderate‐to‐vigorous physical activity a week, had a waist circumference >98 cm, did not have a prior history of cardiometabolic disease, not taking medication, and cigarette non‐smokers. All participants were instructed to maintain their habitual physical activity throughout the course of the study to ensure that the prescribed SIT was additive to their lifestyle. All experimental procedures were approved by both the Health Sciences Human Research Ethics Board of Queen's University and the Health Science Research Ethics Board of Brock University. Verbal and written explanation of the experimental protocol and associated risks were provided to all participants prior to obtaining written informed consent.

### 2.2 Study design

Participants completed 4 weekly sessions of SIT for 4 weeks. Pre‐ and post‐training testing was completed in the week preceding (PRE) and the week following SIT and included the following: (1) a fasted venous blood draw for analysis of serum cytokine and sclerostin concentrations, (2) a fasted adipose tissue biopsy for the determination of adipocyte cell size changes and molecular adaptations to SIT, (3) anthropometric and habitual exercise assessment, and (4) an incremental cycling test to exhaustion for the determination of VO_2peak_.

### 2.3 Pre‐ and post‐training sampling

All pre‐ and post‐training tests followed identical procedures and involved 1 laboratory visit. Post‐training testing was completed ~72 h following the last training session of week 4. Participants reported to the Queen's Muscle Physiology laboratory between 08:00 and 09:00 the morning of testing following a 12‐h overnight fast and abstaining from exercise, caffeine, drugs, and alcohol 24 h prior to testing. Upon arrival, a fasted blood draw was performed from the medial cubital vein using 21G needles and collected in sterile BD Vacutainer^®^ tubes (BD Diagnostics). Blood samples were left at room temperature to clot for 30 min before being centrifuged for 15 min at 1000*g*. Samples were aliquoted and stored at −80°C until analysis.

Adipose tissue biopsies were performed as previously described (Williams et al., 2014). Briefly, biopsies were taken with a Bergstrom needle following local anaesthetization (2% lidocaine) and taken from the abdomen with an incision made ~5 cm lateral to the umbilicus. Approximately 50 mg of adipose tissue was immediately placed in Krebs Ringer Bicarbonate buffer with 4% bovine serum albumin (BSA) and collagenase and incubated for 1 h for isolation of adipocytes and determination of adipocyte size (see below). The remaining tissue was immediately blotted, snap‐frozen in liquid nitrogen, and stored at −80°C until future analysis.

Following the blood draw and biopsy, participants were fed a standardized breakfast that consisted of a toasted bagel (181 kcal; 1 g fat, 36 g carbohydrate, 7 g protein) with 15 g of cream cheese (44 kcal; 4 g fat, 1 g carbohydrate, 1 g protein), and 250 ml of Minute Maid orange juice (56 kcal; 0 g fat, 13 g carbohydrate, 1 g protein). This was followed by measurement of anthropometric values (height [cm], weight [kg], and waist circumference [cm]) and the VO_2peak_ test.

### 2.4 VO_2peak_ test

Thirty minutes after consumption of the standardized breakfast, participants completed a VO_2_max test on a stationary cycle ergometer (Monark Ergomedic 874E, Monark Sports & Medical) using a step protocol that involved incremental increase in Watt Resistance (WR) every minute. Briefly, participants performed 5 min of non‐resisted cycling, followed by a step increase to 80 W for 1 min and subsequent increases in work rate of 25 W per min until participant reached volitional fatigue, or when the participant was unable to maintain a minimum cadence of 70 RPM (Edgett et al. 2013). Gas exchange and heart rate were measured continuously using a metabolic cart (Moxus AEI Technologies). VO_2peak_ was recorded as the highest 30‐s average value obtained during the last stage of the test.

### 2.5 Training protocol

Training was performed as previously described (Raleigh et al., 2018). Briefly, participants completed 4 supervised exercise sessions of SIT per week for 4 weeks, for a total of 16. All training sessions were performed on a mechanically braked cycle ergometer (Monark, Ergomedic 874E) and consisted of a 5‐min warmup (non‐resisted cycling), followed by eight 20‐s intervals targeting 170% of WR at VO_2peak_, separated by 10 s of rest, for a total of 9 min of cycling. Participants were instructed to maintain a cadence of 80 RPM throughout all training sessions, including warm‐up, and non‐resisted rest periods between intervals.

### 2.6 Serum analysis

Serum sclerostin, TNF‐α, and IL‐6 analyses were run in duplicate and analyzed by Quantikine enzyme‐linked immunoassay (ELISA) kits according to manufacturer's instructions (CAT#MSST00 for sclerostin, CAT#HSTA00E for TNF‐α, and CAT#HS600C for IL‐6, R&D Systems).

### 2.7 Adipocyte isolation and determination of cell size

The Krebs Ringer Bicarbonate Buffer was prepared as per manufacture directions (Sigma‐Aldrich). The morning of the biopsy, 4% BSA and 5mg of collagenase (type I, lot no, 81C‐0080, Sigma) were added to the Krebs‐Ringer Buffer and subsequently 50 mg of collected adipose tissue was incubated at 37°C for 1 h. Following incubation, 50 μl of the supernatant was pipetted using a siliconized pipette tip (Bio Plas) and placed on a microscope slide for analysis. Adipocytes were visualized using an AmScope microscope and imaged with a SLR/DSLR Camera adapter and a Nikon D90 camera. A photo was taken of a set scale of 1000 µm at the same magnification for each photo of adipocytes to allow for calibration of images during the determination of the cell size. A 440 × 445 pixels rectangle was drawn on the image and using ImageJ software (ImageJ 1.48v, National Institutes of Health, Java 1.6.0_65 (32‐ bit)) and the diameter of each adipocyte within the rectangle was measured. One investigator analyzed three images from each visit and the mean adipocyte cell size derived from all three images was used as the average adipocyte size for each participant at each time point. The collagenase digestion method is a widely used methods to assess adipocyte cell size as it can easily differentiate lipid droplets based on their appearance due to the absence of a stained plasma membrane (Svensson et al., 2018). It has been validated against other methods and correlates well with total/regional adiposity (Laforest et al., 2017). Importantly, this method is considered gentle and the chances of adipocyte rupture (leading to the presence of lipid droplets) is minimal if centrifugation is avoided (Smith et al., 1972).

### 2.8 Adipose tissue homogenization

Subcutaneous adipose tissue biopsies were homogenized (FastPrep^®^, MP Biomedicals) in 3× volume per mg weight of tissue of NP40 Cell Lysis Buffer (CAT# FNN0021, Life Technologies) supplemented with 3x the recommended volume of phenylmethylsulfonyl fluoride (PMSF) and protease inhibitor (PI) cocktail (CAT# 7626‐5G and P274‐1BIL, respectively, Sigma). The homogenized samples were placed on ice to reduce the foam due to homogenization for 30 min. Samples were then centrifuged at 4°C for 15 min at 16,000*g*, after which the infranatant was collected, and protein concentration was determined using a Bicinchoninic acid assay (CAT # B9643, Sigma‐Aldrich; CAT # BDH9312, VWR). The samples were prepared to contain equal concentrations (1 μg/μl) of protein in 1× Laemmli buffer and placed in a heating block at 100°C for 10 min to boil. Samples were cooled and stored at −80°C for future analysis.

### 2.9 Immunoblotting

For all immunoblots, 15 µg of protein were loaded, except for UCP1, where 3 µg of brown adipose tissue was loaded as a positive control. Proteins were resolved on 10% TGX fast cast gels (CAT# 1610173, Bio‐Rad) for 30 min at 250 V and then were then semi‐dry transferred onto a polyvinylidene difluoride membrane at 1.3 A and 25 V for 7 min (Bio‐Rad Trans‐Blot^®^ Turbo™ Transfer System). Membranes were blocked in Tris buffered saline/0.1% Tween 20 (TBST) with 5% non‐fat powdered milk for 1 h at room temperature. The appropriate primary antibody (1:500–2000 ratio dilution in 5% milk) was then applied and left to incubate on a shaker, at 4°C overnight. Following primary incubation, the membrane was washed with TBST 3 × 5 min and then incubated with the corresponding secondary antibody conjugated with horseradish peroxidase (anti rabbit CAT#HAF008, goat CAT#CAF109, and mouse CAT#HAF 007 IgG—1:2000 dilution in 5% milk, R&D Bio‐Techne) for 1 h at room temperature. Signals were detected using either Clarity™ Western chemiluminescent substrate or SuperSignal™ West Femto maximum sensitivity chemiluminescent substrate (CAT#170‐5061, Bio‐Rad, or CAT#34095 ThermoFisher Scientific, respectively) and were subsequently quantified by densitometry using a Bio‐Rad ChemiDoc™ Imaging System. Each membrane was stained with Ponceau S to confirm equal protein loading and used to normalize. Densitometry analysis was done using ImageLab Software (Bio‐Rad). Antibodies against total STAT3 (CAT# 8768S), phosphoSTAT3‐Tyr705 (CAT#9138S), total JNK (CAT# 9252S), phosphoJNK‐Thr183/Tyr185 (CAT# 4671S), total ERK (CAT#4695S), phosphoERK‐Thr202/Tyr204 (CAT# 9101S), total GSK3β (CAT#12456S), phosphoGSK3β‐Ser9 (CAT#9336S), total β‐catenin (CAT#8480S) were purchased from Cell Signalling. Antibodies against UCP1 (CAT# AB10983) and cytochrome c (CAT#AB76237) were purchased from abcam. PGC‐1α (CAT#AB3242) was purchased from Millipore and COXIV (CAT#A‐6404) was purchased from ThermoFisher Scientific. Sclerostin antibody was purchased from R&D Systems, Inc. (CAT#MAB1406‐SP).

### 2.10 Statistics

Comparisons for all variables were examined with paired two‐tailed *t*‐tests with an α set at 0.05 using GraphPad Prism 9. Data are presented as means ± standard deviation (SD).

## 3 RESULTS

VO_2peak_ increased while there were no anthropometric changes following 4 weeks of SIT. All participants completed all training sessions (100% compliance); however, there was one participant who missed a post‐SIT adipose tissue biopsy, dropping our sample size to 7. VO_2peak_ increased following 4 weeks of SIT (*p* = 0.03, +5.1% from pre‐ to post‐SIT) (Table 1). There was no change in body mass (*p* = 0.7, +0.4% from pre‐ to post‐SIT), body mass index (*p* = 0.7, +0.6% from pre‐ to post‐SIT), waist circumference (*p* = 0.9, +0.1% from pre‐ to post‐SIT), or adipocyte cross‐sectional area (*p* = 0.2, −0.2% from pre‐ to post‐SIT) following 4 weeks of SIT (Table 1).

**TABLE 1:** Anthropometric, aerobic capacity, and adipocyte size response to 4 weeks of SIT

Measure	Pre‐SIT	Post‐SIT	Δ	*p*‐value
Age (y)	25.1 ± 4	—	—	—
Body mass (kg)	113.5 ± 13	113.9 ± 15	+0.4	0.7
BMI	34.7 ± 4	34.9 ± 4	+0.2	0.7
Waist circumference (cm)	112.4 ± 12	112.5 ± 11	+0.1	0.9
VO_2peak_ (ml·kg^−1^·min^−1^)	35.6 ± 7	37.4 ± 9	+1.8	0.03*
Adipocyte cross‐sectional area (µm)	91.3 ± 7	91.1 ± 7	−0.2	0.2

Note

Data is presented as means ± SD. Δ is presented as mean differences of raw values.

* Indicates a significant difference post‐SIT from baseline. Paired samples *t*‐tests were used, and significance was accepted at *p* ≤ 0.05.

### 3.1 Sclerostin content decreased and Wnt/β‐catenin signaling increased within scWAT following 4 weeks of SIT

**FIGURE 1:**
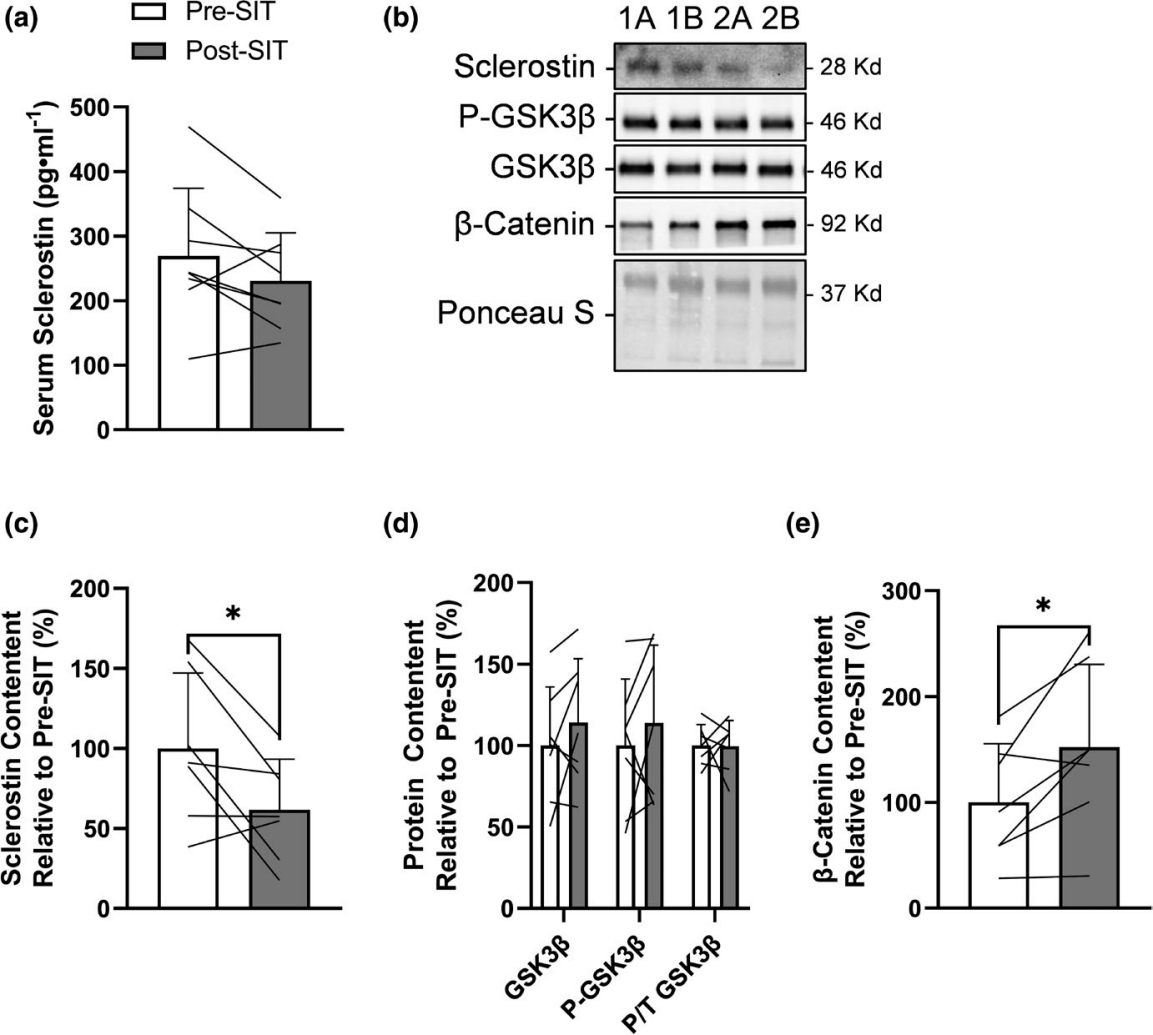
Response of serum sclerostin and Wnt/β‐catenin signaling within scWAT to 4 weeks of SIT. (a) Serum sclerostin response to SIT. (b) Representative immunoblots of sclerostin, phosphorylated and total GSK3β, β‐catenin, and total protein loading control (Ponceau S) at pre‐SIT (labeled “a”) and post‐SIT (labeled “b”) with estimated molecular weights on the right. These representative samples are the same two participants (“1” and “2”) in sequence across proteins that highlight their response from pre‐ to post‐SIT. scWAT (c) sclerostin content, (d) total and phosphorylated GSK3β, and (e) total β‐catenin response to SIT relative to pre‐SIT. Bar charts have black lines that represent individual responses to SIT with group means presented as white (pre‐SIT) and gray (post‐SIT) bars ± SD. Paired *t*‐tests were used to examine changes pre‐ to post‐SIT

To assess if sclerostin was responsive to SIT, we examined peripheral and scWAT sclerostin content, as well as scWAT Wnt signaling components to see if there was a parallel response. There was no change in resting serum sclerostin from pre‐ to post‐SIT (*p* = 0.1, −15% from pre‐ to post‐SIT, 5/7 showed a decrease) (Figure 1a). Representative immunoblots for sclerostin, GSK3β, and β‐catenin are presented in Figure 1b. Specifically, as shown in 1b and 1c, scWAT sclerostin content decreased following SIT (*p* = 0.04, −38% from pre‐ to post‐SIT, 6/7 showed a decrease). To further examine if the decrease in sclerostin augmented Wnt signaling within scWAT, we examined serine 9 phosphorylation of GSK3β and β‐catenin content. It is important to note that GSK3β serine 9 phosphorylation is related to growth factor and Akt inhibition of GSK3β’s kinase activity, while conformational changes that lead to the inhibition of GSK3β’s kinase activity without changes in serine 9 phosphorylation is related to canonical Wnt signaling (McManus et al., 2005; Piao et al., 2008). There was no change in GSK3β content or serine 9 phosphorylation status (*p* = 0.9, 0% change from pre‐ to post‐SIT, 3/7 showed an increase) (Figure 1b and d), while there was an increase in β‐catenin content (*p* = 0.03, 52% from pre‐ to post‐SIT, 6/7 showed an increase) within scWAT following SIT (Figure 1b and e).

### 3.2 COXIV protein content increased following 4 weeks of SIT, while there were no changes in other markers of mitochondrial content

**FIGURE 2:**
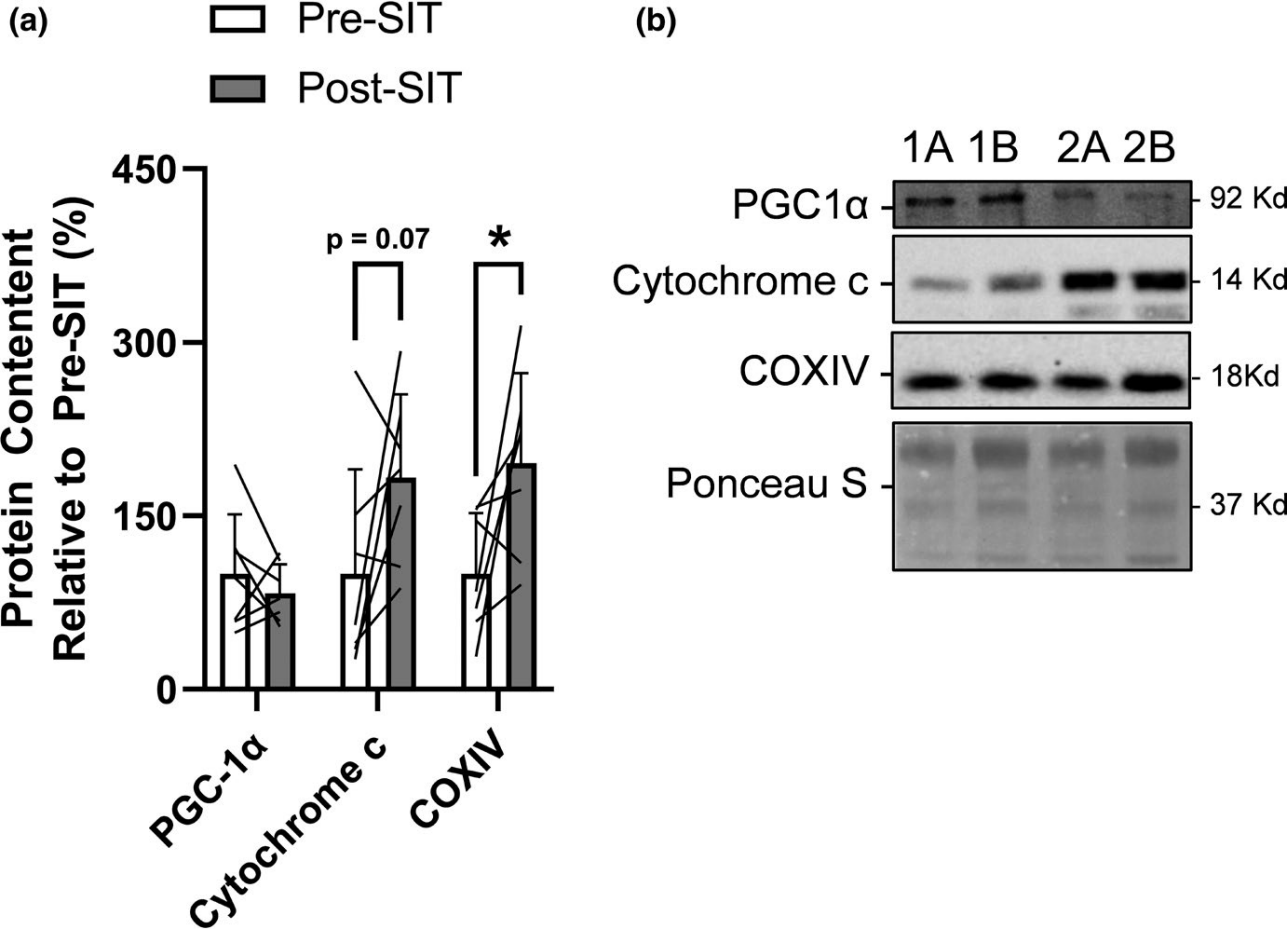
Response of markers of mitochondrial biogenesis and content within scWAT to 4 weeks of SIT. (a) scWAT PGC‐1α, cytochrome c, and COXIV content response to SIT relative to pre‐SIT. Bar charts have black lines that represent individual responses to SIT with group means presented as white (pre‐SIT) and gray (post‐SIT) bars ± SD. Paired *t*‐tests were used to examine changes pre‐ to post‐SIT. (b) Representative immunoblots of mitochondrial proteins (PGC‐1α, cytochrome c, and COXIV) and total protein loading control (Ponceau S) at pre‐SIT (labeled “a”) and post‐SIT (labeled “b”) with estimated molecular weights on the right. These representative samples are the same two participants (“1” and “2”) in sequence across proteins that highlight their response from pre‐ to post‐SIT

Given there is conflicting results on the influence of exercise training on mitochondrial biogenesis in scWAT (no effect (Dohlmann et al., 2018; Hoffmann et al., 2020; Larsen et al., 2015; Pino et al., 2016; Stinkens et al., 2018) vs. increase (Rönn et al., 2014)) and sclerostin/Wnt signaling has been shown to regulate UCP1 content (Kim et al., 2017a) and mitochondrial biogenesis (Mori et al., 2012) in vivo, we immunoblotted for the master regulator of mitochondrial biogenesis, PGC‐1α, components of the electron transport chain, COXIV and cytochrome c, as well as UCP1. Representative immunoblots for PGC‐1α, COXIV, and cytochrome c are presented in Figure 2b. We found no effect of exercise training on PGC‐1α protein content (*p* = 0.41, −27% from pre‐ to post‐SIT, 3/7 showed an increase) or cytochrome c (*p* = 0.07, +83% from pre‐ to post‐SIT, 5/7 showed an increase) (Figure 2a and b) and UCP‐1 was undetectable. However, we found an increase in COXIV (*p* = 0.04, +96% from pre‐ to post‐SIT, 6/7 showed an increase) (Figure 2a and b).

### 3.3 Serum inflammatory cytokine concentrations are reduced with limited effects on inflammatory signaling within scWAT following 4 weeks of SIT

**FIGURE 3:**
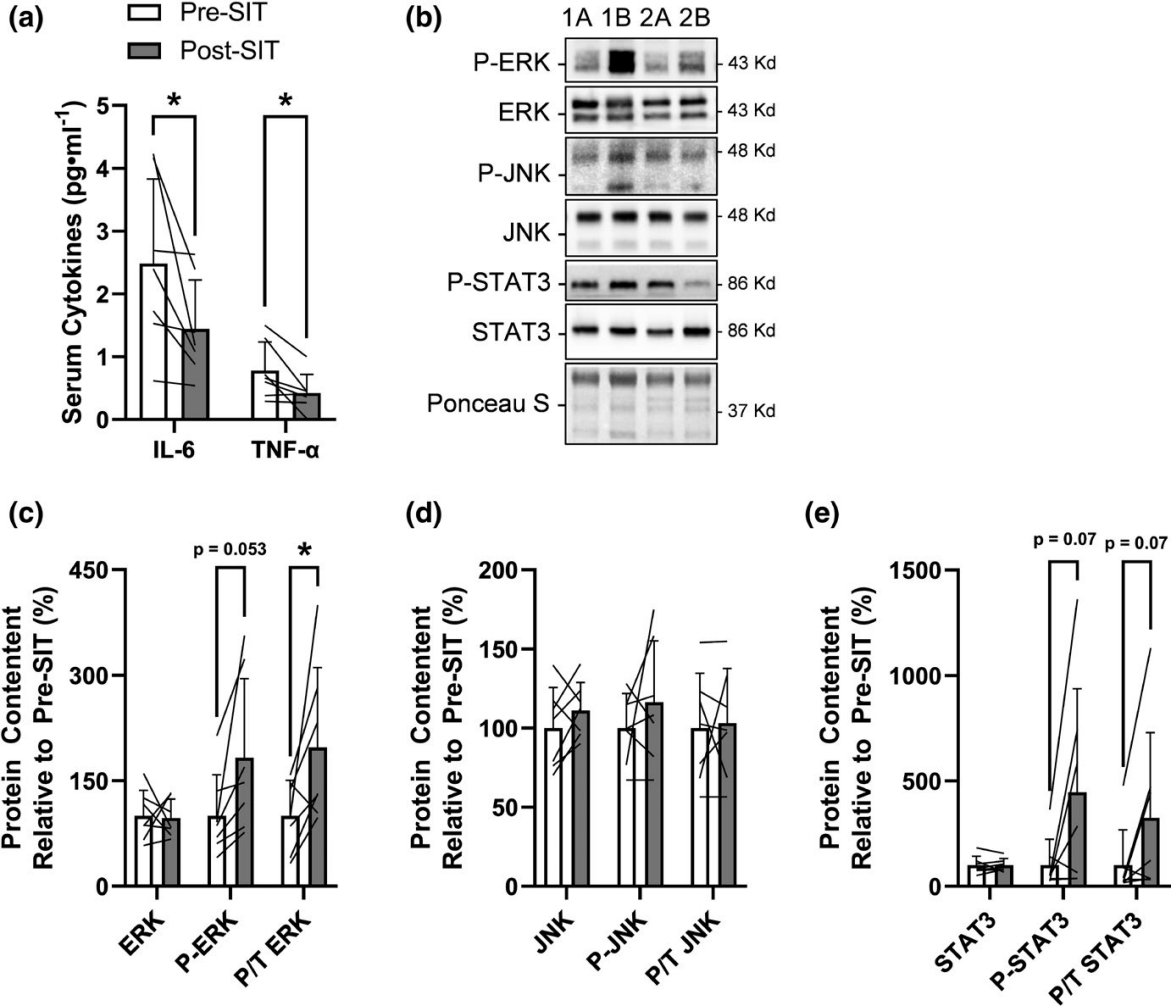
Response of serum inflammatory cytokines and inflammatory signaling within scWAT to 4 weeks of SIT. (a) Serum TNFα and IL‐6 response to SIT. (b) Representative immunoblots for scWAT phosphorylated and total ERK, JNK1/2 and STAT3 and total protein loading control (Ponceau S) at pre‐SIT (labeled “a”) and post‐SIT (labeled “b”) with estimated molecular weights on the right. These representative samples are the same two participants (“1” and “2”) in sequence across proteins that highlight their response from pre‐ to post‐SIT. scWAT (c) phosphorylated and total ERK, (d) JNK, and (e) STAT3 response to SIT relative to pre‐SIT. Bar charts have black lines that represent individual responses to SIT with group means presented as white (pre‐SIT) and gray (post‐SIT) bars ± SD. Paired *t*‐tests were used to examine changes pre‐ to post‐SIT

Obesity is typically characterized by a state of chronic systemic inflammation and exercise is well known to reduce circulating inflammatory cytokines in individuals with obesity and metabolic syndrome (Khalafi & Symonds, 2020). To examine the effectiveness of 4 weeks of SIT on reducing systemic and adipose tissue inflammation we measured circulating markers of inflammation as well as adipose tissue protein markers related to inflammation. Serum TNF‐α (*p* = 0.03, −46% from pre‐ to post‐SIT, 6/7 showed a decrease) and IL‐6 (*p* = 0.05, −42% from pre‐ to post‐SIT, 6/7 showed a decrease) decreased from pre‐ to post‐SIT (Figure 3a). We next wanted to examine downstream inflammatory signaling within scWAT. Representative immunoblots for ERK, JNK, and STAT3 signaling are presented in Figure 3b. There was no change in total content of ERK (*p* = 0.9, −3%), JNK (*p* = 0.3, +11%), or STAT3 (*p* = 0.9, +1%) (Figure 3c–e, respectively). The phosphorylation to total protein content of ERK increased from pre‐ to post‐SIT (*p* = 0.05, +97% from pre‐ to post‐SIT, 6/7 showed an increase), while there was no effect on JNK (*p* = 0.8, +3% from pre‐ to post‐SIT, 2/7 showed a decrease), or STAT3 phosphorylation (*p* = 0.07, −42% from pre‐ to post‐SIT, 5/7 showed an increase) (Figure 3c–e, respectively).

## 4 DISCUSSION

In this pilot study, we found a concomitant reduction in sclerostin content and increased β‐catenin accumulation within scWAT following 4 weeks of SIT, providing novel, observational evidence that sclerostin and Wnt signaling have a role in regulating adipose tissue adaptations to exercise training in overweight adult males. We also observed decreases in peripheral inflammatory cytokines, and increased COXIV, a marker of mitochondrial content, within scWAT following 4 weeks of SIT. Adipose tissue is a critical regulator of whole‐body energy homeostasis and metabolism and is essential in mediating the beneficial effects of exercise training (e.g., improved insulin sensitivity and glucose handling) (Stanford & Goodyear, 2016; Stanford et al., 2015). Examining which molecular/signal transduction pathways within adipose tissue are responsive to exercise and regulate metabolism and cell fate is essential in understanding mechanisms leading to long term adaptations.

In the present pilot study, we found no significant effect of SIT on circulating sclerostin (−15%, *p* = 0.2), which contradicts the few previous studies reporting a decrease in circulating sclerostin with exercise training in untrained normal weight individuals (Hinton et al., 2017; Janik et al., 2018; Sansoni et al., 2018). For example, men aged ~44 years performing either resistance (2×/week) or plyometric training (3×/week) for 12 months have shown a ~7% reduction in circulating sclerostin (Hinton et al., 2017). Likewise, younger men (~24 years of age) performing all out sprint interval training (3 days/week) for 8 weeks had a ~30% reduction in circulating sclerostin (Sansoni et al., 2018). It is possible, that the non‐significant 15% decrease we report herein might be due to our small sample size, length of training period (e.g., 4 vs. 8 weeks (Sansoni et al., 2018)), or difference in exercise mode. However, we found that sclerostin decreased and Wnt signaling was augmented within scWAT in response to SIT, suggesting a role in mediating adaptations within scWAT with exercise training. An additional consideration for the discrepancy between the sclerostin response in the circulation and scWAT in this study is the methods used to detect sclerostin. Sclerostin is known to travel in the circulation not only as a monomer, but also primarily as a dimer (Hernandez et al., 2014). Examining peripheral sclerostin with ELISA detects both the monomeric and dimeric sclerostin content, thus diluting the effect exercise may have on a specific proteoform (Carbonara et al., 2021) of sclerostin (e.g., its monomer). In contrast, we only analyzed the response of the monomeric form of sclerostin, i.e., the functional form of sclerostin (Kim et al., 2020), within scWAT with the use of immunoblotting, which improves sensitivity in detecting changes in this specific proteoform to our exercise intervention. No previous study has examined the effect of exercise on human adipose tissue sclerostin content, which provides support for further examination of the importance of this response. This preliminary new evidence of increased Wnt signaling within scWAT following SIT in humans is supported by a study examining mice performing voluntary wheel running for 11 days, which showed an increased gene expression of downstream targets of Wnt signaling within scWAT when compared to sedentary control mice (Stanford et al., 2015). In support of the specific role of Wnt signaling, we also found an increase in β‐catenin that was not associated with any changes in GSK3β phosphorylation, suggesting that the increase in β‐catenin was linked to Wnt activation and subsequent dissociation of the destruction complex (Piao et al., 2008). Thus, we propose a role of sclerostin and Wnt signaling in regulating long term adaptations to adipose tissue with exercise training, which requires additional human studies to confirm.

The importance of this role of sclerostin is highlighted in murine models, where inhibition of sclerostin action has led to reduced adiposity, protection against an obesogenic diet, and improved glucose handling and fat oxidation (Kim et al., 2017b, 2019). Additionally, bone‐specific knockout of sclerostin leads to increased adipoprogenitors in the stromal vascular fraction, which is due to their inhibited maturation (Kim et al., 2021). Similarly, Wnt overexpression (Aslanidi et al., 2007; Longo et al., 2004; Wright et al., 2007), either by treatment with a small molecule Wnt activator (Choi et al., 2014) or induced activation of Wnt signaling in adipocyte progenitors (Zeve et al., 2012), has shown to prevent adipose tissue accumulation and improve glucose homeostasis and insulin sensitivity in genetically obese mice (e.g., ob/ob mice) or those fed a high fat diet. Taken together these lines of evidence suggest that Wnt activation within adipose tissue regulates cell fat and metabolism. Our results suggest adaptations in adipose tissue with exercise training may occur through activating Wnt signaling and reduced sclerostin. While there is no reported data on body composition/fat mass responses following treatment with sclerostin neutralizing antibodies (Glorieux et al., 2017; Padhi et al., 2011, 2014), this may be an alternative use for sclerostin neutralizing antibodies. Thus, future clinical trials should examine the response of adiposity to sclerostin neutralizing antibody treatment with and without exercise training in individuals with obesity and metabolic syndrome to separate the exercise induced responses from those of such treatment in humans.

While the main objective of this study was to examine the response of sclerostin and Wnt signaling within scWAT to SIT, a secondary objective was to examine markers of mitochondrial content. This was a natural extension given Wnt signaling promotes adipocyte mitochondrial biogenesis and maximal oxygen capacity in isolated mitochondria and inhibiting Wnt signaling in vivo prevents oxidative metabolism and stimulates maximal adipocyte growth when mice are given an obesogenic diet (Mori et al., 2012). Herein, we found an increase in COXIV, while we observed no change in cytochrome c, PGC‐1α, or UCP‐1. In humans, obesity inhibits mitochondrial biogenesis in subcutaneous adipose tissue (Heinonen et al., 2015), and active individuals tend to have higher mitochondrial content in scWAT compared to sedentary controls (Pino et al., 2016). While improvements in mitochondrial function or content with exercise training in murine models is well established (Stanford & Goodyear, 2016), evidence in humans is less conclusive, showing either an increase in mitochondrial content or function (Dohlmann et al., 2018; Rönn et al., 2014) or no change (Hoffmann et al., 2020; Larsen et al., 2015; Pino et al., 2016; Stinkens et al., 2018) with exercise training. The paucity of data restricts detailed discussion of the influence of participant characteristics or exercise training mode or duration. However, the only studies that have shown increases in mitochondrial function or content within scWAT of humans are those with a longer duration intervention (6 months) (Rönn et al., 2014) or interventions with higher volume or intensity of exercise (Dohlmann et al., 2018), including the present study. Given the role of Wnt signaling in regulating mitochondrial biogenesis, the influence of decreased sclerostin and increased Wnt signaling on mitochondrial biogenesis within scWAT following SIT warrants further investigation.

There are limited studies examining the response of UCP1 to exercise training in human adipose tissue and most studies have looked at gene expression data, which have found that *UCP1* in scWAT either decreases (Brandao et al., 2019), does not change (Norheim et al., 2014; Pino et al., 2016), or increases (Norheim et al., 2014; Otero‐Díaz et al., 2018) if groups (controls vs. patients with pre‐diabetes are collapsed). Two studies have examined UCP1 positive adipocytes with immunohistochemical analysis and have found either no response to exercise training (Tsiloulis et al., 2018) or an increase (Otero‐Díaz et al., 2018). Cold acclimation inhibits GSK3β and subsequently increases UCP1 expression (Markussen et al., 2018). Thus, we speculated that, if UCP1 is detectable and responsive to exercise training, exercise would decrease scWAT sclerostin leading to inhibition of GSK3β and increased UCP1 expression. However, when we examined UCP1 protein content we were unable to detect any bands at UCP1’s theoretical molecular weight, providing further evidence humans have negligible UCP1 content in scWAT and exercise training is not a sufficient stimulus to induce an increase in expression.

We also examined the influence of SIT on circulating cytokines and their associated signaling pathways within scWAT. Serum IL‐6 and TNF‐α significantly decreased post‐SIT (−46% and −41%, respectively). This finding is in agreement with previous clinical studies also showing reductions in pro‐inflammatory cytokines with various modes of exercise training in patients with obesity or metabolic disorders (Khalafi & Symonds, 2020), heart failure (Pearson et al., 2018), multiple sclerosis (Negaresh et al., 2018), as well as cancer survivors and older adults (Khosravi et al., 2019; Sardeli et al., 2018). Interestingly, the reduction in inflammatory cytokines following SIT in our group of men with overweight and obesity occurred despite having no changes in fat mass and having relatively low TNF‐a and IL‐6 at baseline (Todd et al., 2013). This is in contrast to what is typically seen in individuals with obesity, who tend to have higher circulating TNF‐α and IL‐6 that subsequently decrease following interventions that combine diet and exercise that lead to reductions in fat mass (Nicklas et al., 2005). While we saw a reduction in circulating IL‐6 there was a small non‐significant increase (*p* = 0.07) in STAT3 activation in scWAT 72h following 4 weeks of SIT, which may be a result of an increase in the IL‐6 receptor content (Leggate et al., 2012). The importance of IL‐6 signaling and the reductions of adipose tissue to exercise training has been previously highlighted in humans (Wedell‐Neergaard et al., 2019), thus reductions in IL‐6 peripherally with no change in IL‐6 signaling within scWAT may be beneficial. Additionally, the reduction in circulating TNF‐α did not result in reductions in JNK1/2 activation within scWAT following SIT. While TNF‐α appears to be more involved in the mechanism and progression of the metabolic syndrome (e.g., reduced insulin sensitivity through an increase in JNK1/2 activation (Solinas & Becattini, 2016)), IL‐6 is likely only a marker of metabolic syndrome since it has been shown to increase lipolysis and fat oxidation (Petersen & Pedersen, 2005). This is likely why most studies have shown a reduction in TNF‐α and no change to IL‐6. Lastly, we observed an increase in ERK1/2 phosphorylation, which contrasts with our peripheral TNF‐α data. ERK is a multifaceted protein that is involved in several biological functions that include cell proliferation, differentiation, metabolism (e.g., insulin signaling), and transcription, and its signaling can be activated by inflammation as well as several proteins released during exercise (e.g., FGF21 and irisin) (Geng et al., 2019; Zhang et al., 2014). Together, our results indicate peripheral reductions in circulating cytokines do not correspond to changes in their associated signaling pathways in scWAT (i.e., activated independent of inflammation). This suggests there is alternative signaling molecules (e.g., proteins released during exercise) inducing the activation of ERK1/2, which may be related to exercise induced adaptations within adipose tissue (Knudsen et al., 2014; Stanford & Goodyear, 2016; Wedell‐Neergaard et al., 2019; Zhang et al., 2014).

This was an initial pilot study on this topic, and as such, it was limited by the small sample size (*n* = 7), lack of a non‐exercising control group, and the relatively short duration of training (4 weeks). Additionally, the lack of a lean comparison group prevents conclusions on the magnitude of response among participants of different body composition.

## 5 CONCLUSION

In this pilot study we found a reduction in sclerostin and an increase in β‐catenin content within scWAT following 4 weeks of SIT, suggesting a potential role of sclerostin and Wnt signaling in mediating adaptations within scWAT to exercise training in humans. We also found an increase in one marker of mitochondrial content (COXIV) and a downregulation in circulating inflammatory cytokines (TNF‐α and IL‐6) that did not translate to a reduction in their associated signaling cascades within scWAT. Future studies should aim to examine the importance of exercise induced changes in sclerostin content and Wnt signaling to the long‐term adaptations to adipose tissue.

## CONFLICT OF INTEREST

The authors have no conflict of interest to declare.

## ETHICS STATEMENT

This research was approved by our Universities Research Ethics Board and we obtained written and oral consent from each participant.

## Supporting information



Figure S1
