# Supplementary figures and images for: Subcutaneous adipose tissue sclerostin is reduced and Wnt signaling is enhanced following 4‐weeks of sprint interval training in young men with obesity
Source: Physiol Rep. 2022 Mar 21;10(6):e15232. doi: 10.14814/phy2.15232 (PMC8935536; doi:10.14814/phy2.15232)

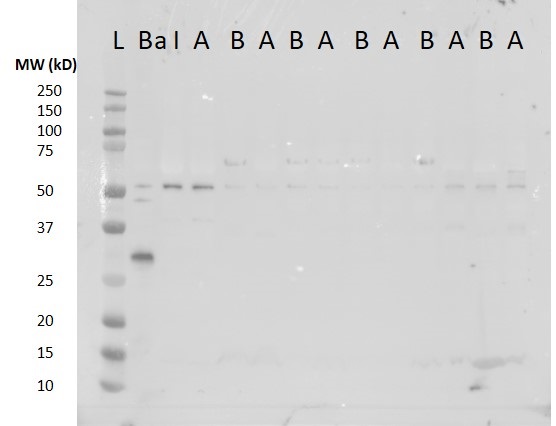

Supplement: Supplementary file 1 — Figure S1 [file PHY2-10-e15232-s001.jpg]
